# Classification of human enteric neurons

**DOI:** 10.1007/s00418-021-02002-y

**Published:** 2021-06-25

**Authors:** Axel Brehmer

**Affiliations:** Institute of Anatomy and Cell Biology, Friedrich-Alexander Universität Erlangen-Nürnberg, Krankenhausstr. 9, 91054 Erlangen, Germany

**Keywords:** Dogiel, Enteric nervous system, Neuroanatomical terminology, Stach

## Abstract

Major advances in our understanding of the functional heterogeneity of enteric neurons are driven by the application of newly developed, innovative methods. In contrast to this progress, both animal and human enteric neurons are usually divided into only two morphological subpopulations, “Dogiel type II” neurons (with several long processes) and “Dogiel type I” neurons (with several short processes). This implies no more than the distinction of intrinsic primary afferent from all other enteric neurons. The well-known chemical and functional diversity of enteric neurons is not reflected by this restrictive dichotomy of morphological data. Recent structural investigations of human enteric neurons were performed by different groups which mainly used two methodical approaches, namely detecting the architecture of their processes and target-specific tracing of their axonal courses. Both methods were combined with multiple immunohistochemistry in order to decipher neurochemical codes. This review integrates these morphological and immunohistological data and presents a classification of human enteric neurons which we believe is not yet complete but provides an essential foundation for the further development of human gastrointestinal neuropathology.

## Prologue: from past to present

The earliest approaches to identify nerve structures in the gut resulted in the description of different ganglionic enteric nerve networks (Meissner 1857; Auerbach 1862; Schabadasch 1930) and the subsequent distinction of enteric neuron types (Dogiel 1899). The latter phase in particular was embroiled in decades of dispute between followers of two rival theories concerning the basic structure of the nervous system, the reticular versus the neuron theory, the latter mainly based on the works of Ramón y Cajal (Garcia-Lopez et al. 2010). This conceptual discord was only decided in favor of the neuron theory in the 1950s by electron microscopy (Clarke and O’Malley 1996).

The Russian histologist Dogiel was a rather inconsistent follower of the reticular concept because he distinguished neural processes into long and short ones, namely axons and dendrites (Brehmer et al. 1999a). Although his descriptions of three neuron types contained the hidden potency for further development, “collapsing of the classifications” (Furness and Costa 1987) led to a simplified morphological two-type concept (Brehmer et al. 1999a). For numerous contemporary researchers in the field of the enteric nervous system (ENS), the distinction between *Dogiel type II* and *Dogiel type I* neurons is mainly useful for separating primary afferent from all other enteric neurons (Nurgali 2009; Carbone et al. 2014; Fung and Vanden Berghe 2020; Spencer and Hu 2020; Smolilo et al. 2020; Yuan et al. 2021). The occasional identification of non-Dogiel type I or II but *filamentous neurons* (Furness et al. 1988; Carbone et al. 2014) indicated both the possibility and the need for a more solid morphological classification reminiscent of Dogiel’s original tree-type system.

### Three original types of Dogiel

Although Dogiel (1899) reported that he also considered submucosal neurons, his three types are obviously concerned with myenteric neurons (based on specimens from human infants, guinea pigs and other mammals). Of his depictions, only samples of type I and II neurons are derived from humans (Brehmer 2006).

*Type I neurons* had one axon and up to 20 dendrites which were, in simple terms, short with broad or lamellar endings. Axons of these neurons were observed to run through adjacent ganglia, occasionally into the muscle coat.

*Type II neurons* had one axon and up to 16 long dendrites leaving the ganglion of origin and resembling axons—a distinctive ambiguity (see below).

*Type III neurons* had one axon and up to 10 (seldom more) dendrites which were many times longer than those of type I neurons, ramified and had tapering endings within the ganglion of origin (in contrast to the processes of type II neurons). Only two examples derived from guinea pig large intestine were depicted by Dogiel (1899).

Following the categories of the recent International Neuroanatomical Terminology (FIPAT. Terminologia Neuroanatomica. 2017), all these Dogiel types of neurons would be called *multipolar neurons* displaying one (long or short) axon and several dendrites.

### Additional criteria of Stach

Based mainly on investigations on silver-impregnated whole mounts of the pig small intestine, two general, conceptual advances as to morphological classification schemes were achieved by Stach in the 1980s.

First, he strictly considered the *combination* of two (at first glance independent) morphological features, namely the dendritic architecture *and* the axonal course in Dogiel’s types. This resulted in the clear distinction between (pig) type I neurons with short, lamellar dendrites, *and* orally running axons as well as (pig) type III neurons with long, branched, tapering dendrites *and* anally running axons (Stach 1980, 1982a). This conceptual progress forced a numerical extension beyond Dogiel’s three types. Stach type IV neurons (in the pig) had short, tapering dendrites *and* axons running vertically towards the mucosa (Stach 1982b; Brehmer et al. 1999b).

Secondly, Stach specified the generally used term “multipolar” for neurons displaying numerous processes. On the one hand, “multipolar type I, III, IV neurons” (and also further types V, VI: Stach 1985, 1989) were termed *multidendritic uniaxonal neurons.* On the other hand, “multipolar type II neurons” were characterized as *multiaxonal neurons* (Stach 1981; there are both non-dendritic and dendritic ones, see below). This distinction within the category “multipolar neurons” is not considered in the recent neuroanatomical terminology (FIPAT. Terminologia Neuroanatomica. 2017).

### Enteric neuron classifications in different mammalian species

Our conceptual understanding of enteric circuits in general is derived mainly from the guinea pig (Furness and Costa 1987; Furness 2006). That is, intrinsic afferent neurons, several types of ascending and descending interneurons or various muscle or mucosal motor neurons were first identified in this species. The immunohistochemical detection of the presence or absence of neuronal substances (i.e., the chemical coding of enteric neurons) became an effective and easily applicable tool for distinction of enteric neuron types in the guinea pig and, subsequently, in other species. In contrast, morphological correlates of this chemical and functional heterogeneity were hardly searched for.

Detailed knowledge on neurochemical classes of neurons is available from various gut segments of the guinea pig, e.g. the small intestine (Costa et al. 1996; Brookes 2001), the colon (Lomax and Furness 2000) and the stomach (Schemann et al. 2001). Considerable data also exist from the mouse (Sang et al. 1997; Nurgali et al. 2004; Qu et al. 2008; Mongardi Fantaguzzi et al. 2009), the rat (Sayegh and Ritter 2003; Mitsui 2010), the pig (Brown and Timmermans 2004; Brehmer 2006; Petto et al. 2015; Mazzoni et al. 2020) and other mammals (Freytag et al. 2008; Chiocchetti et al. 2009; Noorian et al. 2011).

It was recognized early on that the transfer of single findings from one species to another (including human) is hampered by species differences. Furthermore, the concept “one neuron–one function” has been refuted. Intrinsic primary afferent neurons (IPANs, see below) can also be regarded as interneurons (Wood 1994; Furness et al. 1998), and non-IPANs have also been shown to be (mechano-)sensory (Spencer and Smith 2004; Spencer and Hu 2020), even in the human ENS (Kugler et al. 2015).

Because of the restricted access to human tissues, our knowledge of human enteric morphochemical classes is much more limited than that of some animal species. Therefore, the following assignment of functions to morphochemical phenotypes remains partly putative and certainly incomplete. The aim of this review is to integrate findings of morphological and immunohistological features of human enteric neurons and to stimulate further search for both physiological features and pathohistological alterations in the human ENS. It has been shown that both dendritic architecture (Brehmer et al. 2001) and chemical coding of neurons (Schemann and Neunlist 2004) may change under experimental and pathological conditions, respectively.

## Structure of human myenteric neurons

Recent attempts to identify and characterize the structure and main functions of human enteric neuron populations followed two methodical approaches.

One method aimed at the representation of dendrites and proximal axonal segments of neurons (Table 1). Classically, this has been achieved by silver impregnation (Stach 1980, 1989; Stach et al. 2000). Presently, this capricious method is being replaced by immunostaining with cytoskeletal markers which depicts neurons almost equivalently (Vickers and Costa 1992; Brehmer et al. 2002b, 2004a) but is a more reliable and combinable staining method. We used neurofilaments (NF; Brehmer et al. 2004a) in human myenteric and peripherin (PERI; Kustermann et al. 2011) in human submucosal neurons. These markers represent the morphology of the cytoskeleton but not that of the whole, membrane-covered neuron. They allowed us, by co-staining with other neuronal markers, to differentiate neurons based on their morphochemical phenotype. That is, neurons displaying different dendritic architectures and, simultaneously, different chemical codes were distinguished from each other. In this context, application of the panneuronal marker human neuronal protein HuC/D (HU) enabled estimation of proportions of enteric subpopulations (Ganns et al. 2006).

**Table 1:** Summary of human myenteric neuron types based on their morphological properties observable after immunostaining for neurofilaments (NF)

Myenteric neuron type	Basic morphological description	Axon projection Dendritic architecture	Chemical coding	Main function? (comments)
Type II	Pseudouni- to multiaxonal	A: partly circumferential, partly to mucosa D: non-dendritic	ChAT/ CALR/SOM/SP	IPAN? (usual subtype)
		A: not known D: long, branched, tapering	ChAT?	IPAN? (infrequent subtype)
Stubby type I	Uniaxonal Dendritic	A: mostly orally D: short, radially arranged: “stubby, lamellar”	ChAT/ENK/SP±	Ascending interneuron? Excitatory motor neuron?
Spiny type I	Uniaxonal Dendritic	A: mostly anally D: short, radially arranged: “spiny, thorny”	nNOS/VIP/GAL± ChAT/nNOS/VIP±	Inhibitory motor neuron? Descending interneuron?
		D: with few additional main dendrites	nNOS/VIP	(Only in upper small intestine)
Hairy type I	Uniaxonal Dendritic	A: to mucosa? D: short, thin, radially arranged; as a whole: “hairy”	ChAT/VIP (determined only in stomach)	Mucosal motor neuron? (Stach type IV in pig?)
Type III (in small intestine)	Uniaxonal Dendritic	A: anally? D: long, radially arranged, branched, tapering	ChAT/CALB/ CALR±/SOM±	Interneuron? (Stach type III in pig? Filamentous neuron in guinea pig?)
Type V (in upper small intestine)	Uniaxonal Dendritic	A: anally D: mostly single stem process with long, branched, tapering dendrites	ChAT/SOM±	Interneuron? (Stach type V in pig?)
Non-specific neurons	Uniaxonal (inconspicuous dendritic?)	A,D: not specified / A,D: not specified	ChAT / nNOS	

Roman numerals I–III refer to Dogiel’s and IV–V to Stach’s types. For details and references see text

The other method deciphered target tissues of axonal projections by tracing techniques (most successful with the carbocyanine tracer DiI; Wattchow et al. 1995, 1997; Porter et al. 1997, 2002; Humenick et al. 2019, 2021). These studies demonstrated projection distances for motoneurons of about 1 to 2 cm (for ascending and descending pathways to circular and longitudinal muscle, respectively) as well as for interneurons of about 4 to 7 cm (for ascending and descending interneurons, respectively).

Since both approaches were combined with multiple immunohistochemistry for neuronal substances, correlation of the results is possible to some degree.

### Dogiel type II neurons

Also, with regard to the entire human (and mammalian) nervous system, Dogiel type II neurons (Dogiel 1899) are unique. Already the Russian histologist was apparently uncertain in his own interpretation when distinguishing between one axon and (up to) 16 dendrites in this type (Brehmer et al. 1999a). Stach (1981) introduced the term “multiaxonal” because, neurohistologically, all processes of these neurons *look like axons.* Hendriks et al. (1990) showed that all these processes conduct action potentials; thus, electrophysiologically, they *behave like axons*. Altogether, they were characterized in the guinea pig small intestine as intrinsic primary afferent neurons (IPANs), the only ones known so far in a peripheral organ (Furness et al. 1998). Individual features of the equivalent neurons in various species were shown to differ. The electrophysiological afterhyperpolarization (AHP) phenomenon of IPANs in the guinea pig could barely be proven in humans (Brookes et al. 1987), and the chemical coding between IPAN-candidates of other species varied considerably, apart from their common cholinergic phenotype. In the guinea pig, calbindin (CALB) is an effective marker for IPANs (Furness et al. 1990; Song et al. 1994). Calcitonin gene-related peptide (CGRP) is, among other additional markers, immunohistochemically demonstrable in putative IPANs of pigs (Scheuermann et al. 1987; Wolf et al. 2007), mice (Furness et al. 2004; Melo et al. 2020), lambs (Chiocchetti et al. 2006) and rats (Mitsui 2009).

**Fig. 1:**
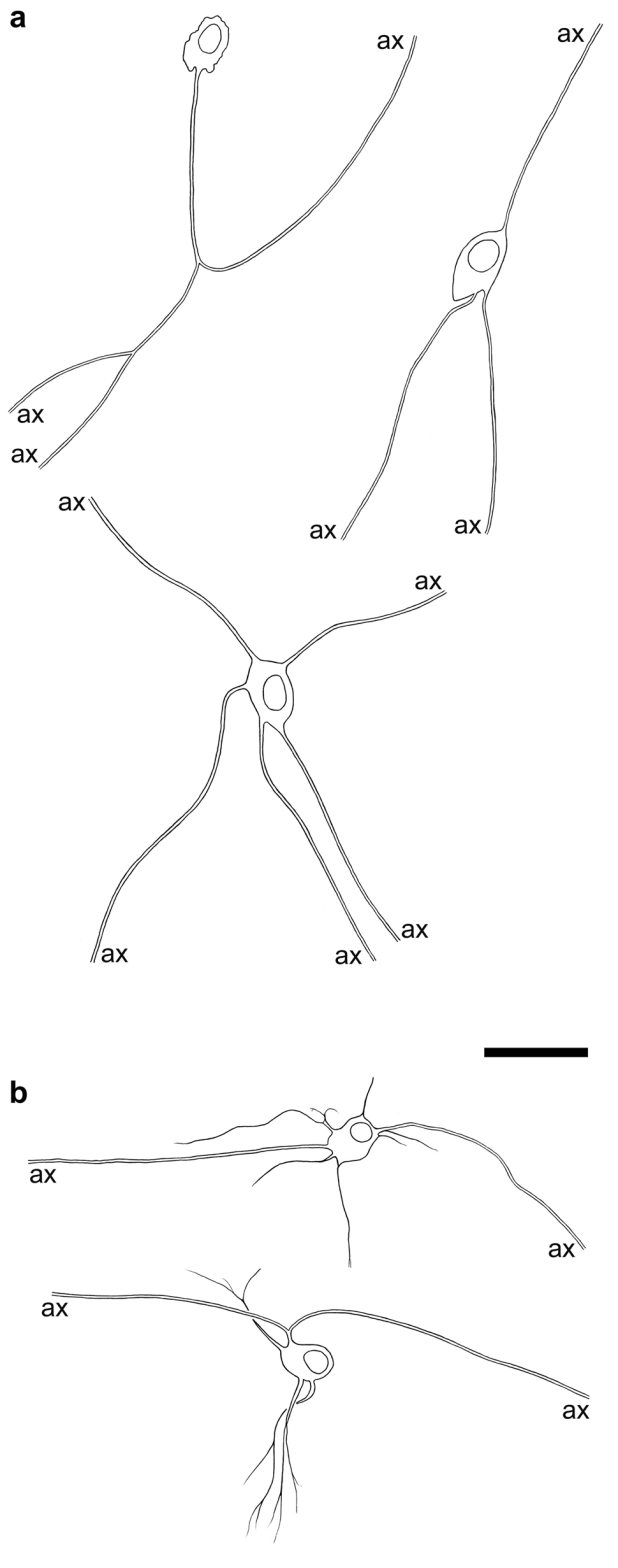
**a** Drawings of three non-dendritic, multiaxonal type II neurons from the human small intestine. **b** Two dendritic, multiaxonal type II neurons from the small intestine. (Axons are illustrated by double lines until their cut ends = ax) Bar = 50 µm

In the human small intestine, Dogiel type II neurons (Fig. 1a), the putative IPANs, amount to about 10% of the whole myenteric neuron population. Immunohistochemical co-labelling of calretinin (CALR), somatostatin (SOM) and substance P (SP) is characteristic for these neurons (Brehmer et al. 2004b; Weidmann et al. 2007), whereas both CALB and CGRP are only detectable in a minority of type II neurons (Brehmer 2007).

In the human stomach, less than 1% of myenteric neurons could be morphologically identified as type II neurons, and most of them were SOM-reactive, with weak or no co-reactivity for CALR (Anetsberger et al. 2018).

In the human colon, co-labelling of CALR and SOM is, similar to the small intestine, highly indicative for type II neurons; however, about one third of CALR+/SOM- neurons and about half of SOM+/CALR- neurons were also NF-reactive, multiaxonal type II neurons (own unpublished observations).

Next to these non-dendritic Dogiel type II neurons there are *dendritic type II neurons* (Fig. 1b). These also have more than one axon and additional dendrites and were originally described in the pig (Stach 1989). In the guinea pig, they were characterized as IPANs (Bornstein et al. 1991; Brookes et al. 1995). In humans, they were occasionally identified in the small intestinal myenteric plexus (Stach et al. 2000) and are non-nitrergic, probably cholinergic neurons (Brehmer 2006).

**Fig. 2:**
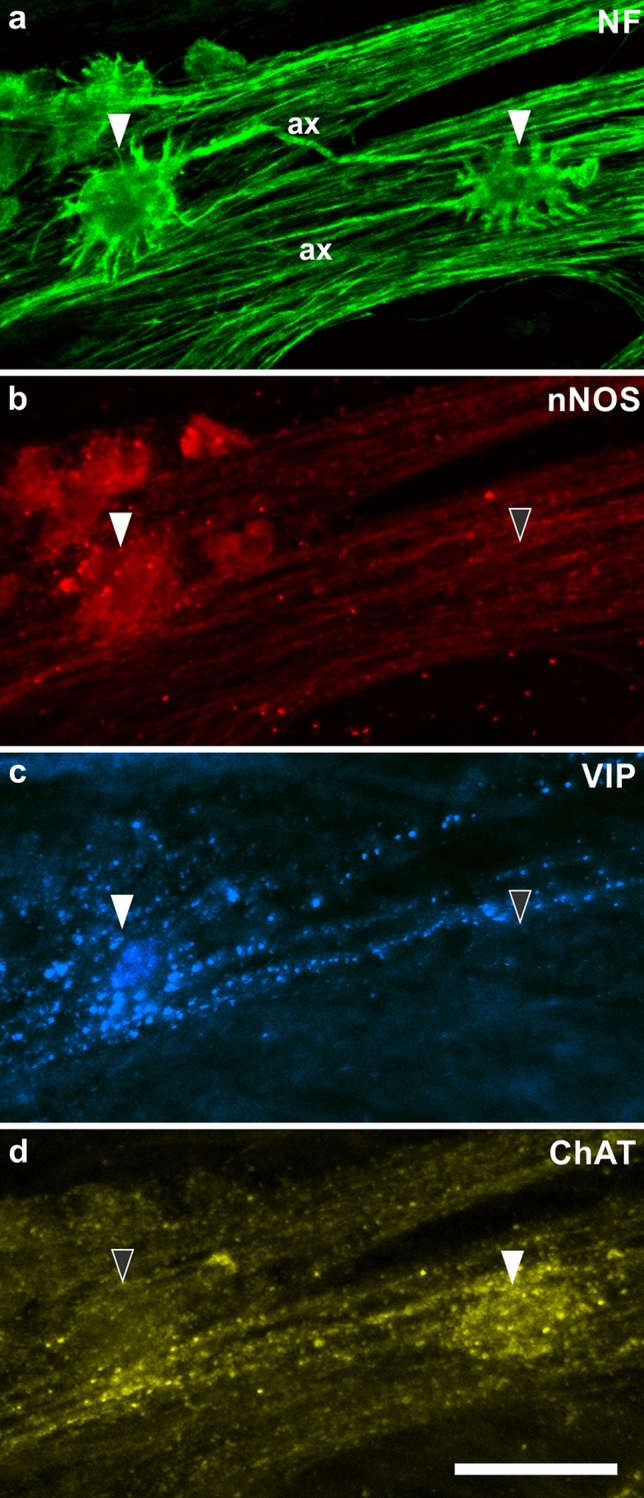
**a** Two “Dogiel type I” neurons (filled arrowheads) stained for neurofilaments (NF). The left one is a spiny neuron, its axon (ax) runs to the right (i.e. anally); the right one is a stubby neuron with an axon (ax) running to the left (i.e. orally). **b** Corresponding demonstration of staining for neuronal nitric oxide synthase (nNOS). The spiny neuron is positive, the stubby one negative (filled and empty arrowhead, respectively). **c** The spiny neuron is co-reactive for vasoactive intestinal peptide (VIP), the stubby neuron is negative (filled vs. empty arrowhead). **d** The spiny neuron is negative for choline acetyltransferase (ChAT), the stubby neuron is positive (empty vs. filled arrowhead). (From ascending colon of a 104-year-old woman; body donated to the Institute of Anatomy) Bar = 50 µm. (Antibodies: NF: Sigma-Merck N0142; nNOS: Novus Biologicals NB120-3511; VIP: Dianova T-5030; ChAT: Merck-Millipore AB144P)

**Fig. 3:**
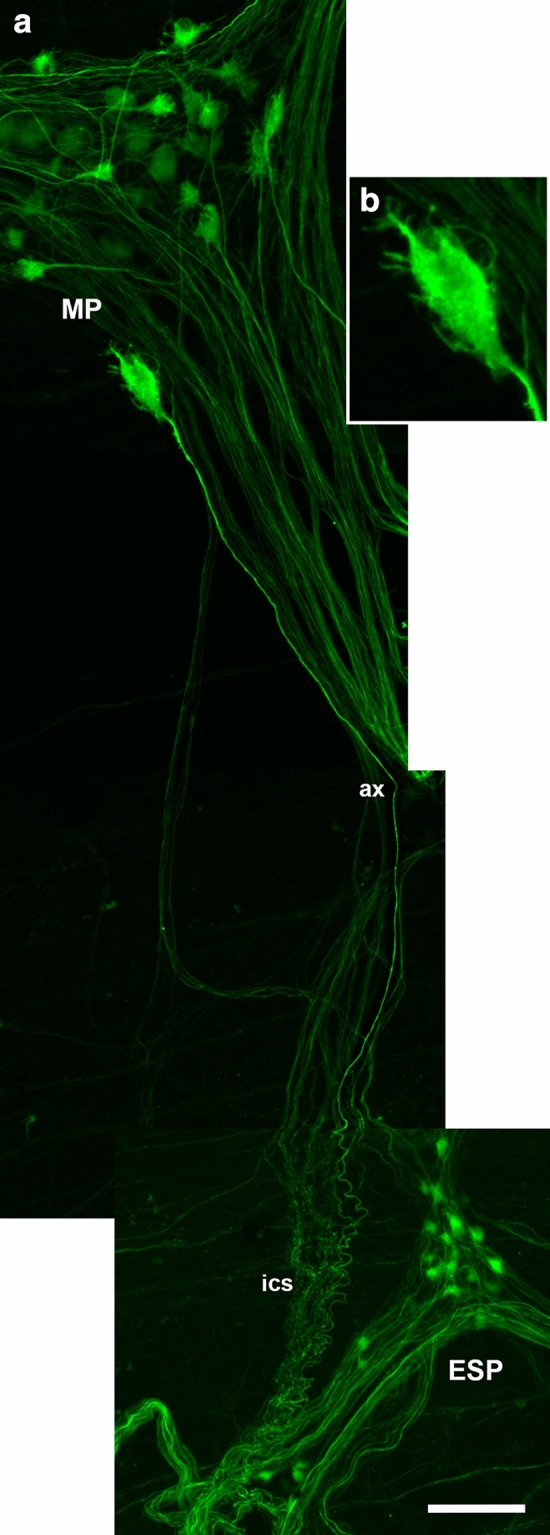
**a** A short-dendritic (“Dogiel type I”) neuron immunopositive for neurofilaments (NF; enlarged in **b**) whose axon (ax) runs from the myenteric plexus (MP) into a typically coiled interconnecting strand towards the external submucosal plexus (ESP). (Myenteric whole mount with adhering remnants of circular muscle strips and submucosal connective tissue, derived from the transverse colonic segment resected from a female patient aged 21 years suffering from colon carcinoma. Composition of four subsequent z-series following the marked axon, each depicted as extended focus image.) Bar = 50 µm

As pointed out above, a key criterion for identifying IPANs in various species is their “Dogiel type II” (i.e. multiaxonal) morphology as opposed to the “Dogiel type I” morphology of neurons with short dendrites and a single axon. Actually, quite different human myenteric neurons are distinguished that fit into this overall category (Figs. 2 and 3).

### Stubby type I neurons

**Fig. 4:**
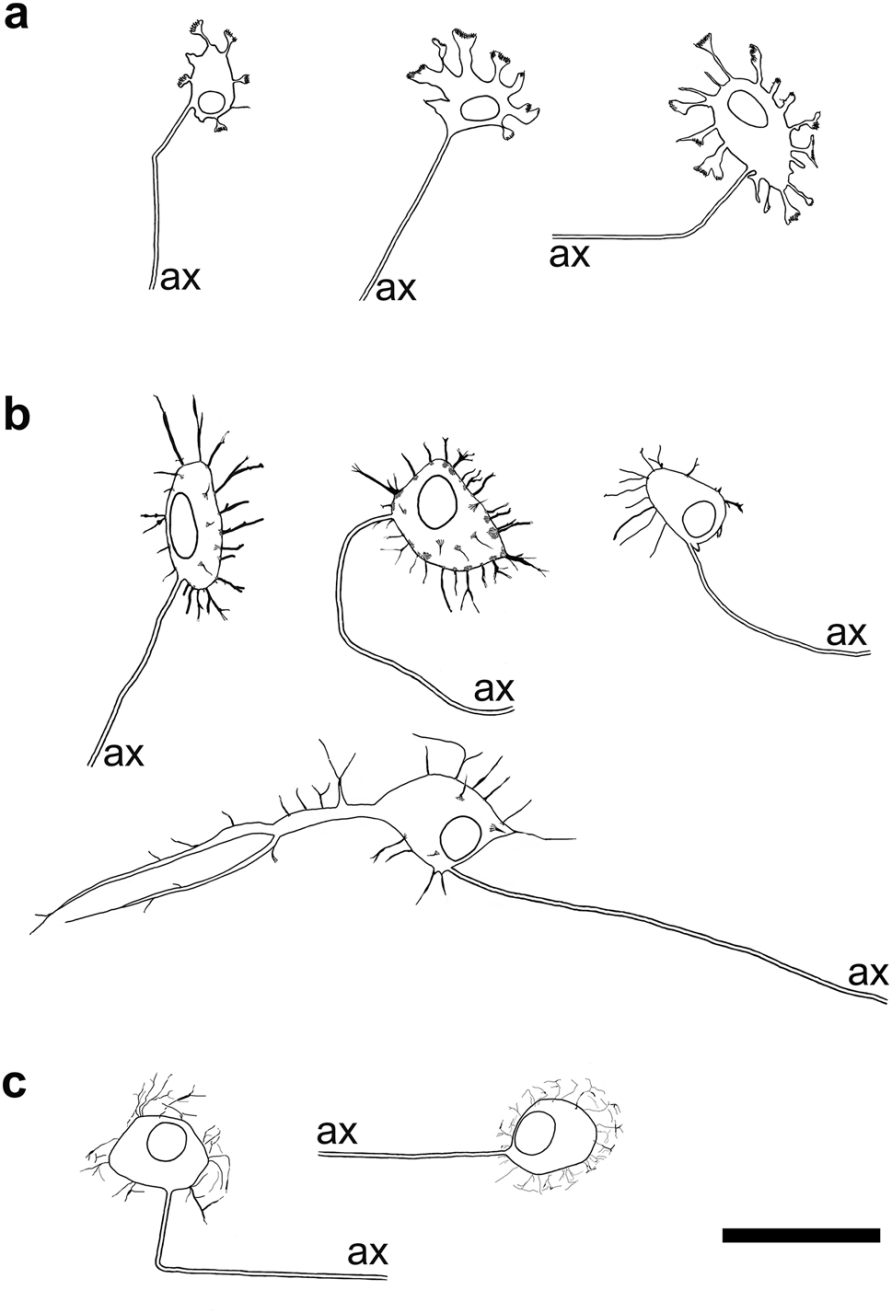
**a** Drawings of three stubby (type I) neurons; the two left ones are from the small intestine, the right one from the large intestine. **b** Four spiny (type I) neurons; the two upper left ones are from the small intestine, the upper right one from the large intestine. The lower one with a main dendrite is from duodenum. **c** Two hairy (type I) neurons from the human stomach. (ax = cut ends of axons) Bar = 50 µm

Due to the shapes of their “short” processes, demonstrated by NF-immunohistochemistry, these human myenteric neurons most likely match Dogiel’s original descriptions of type I neurons (Fig. 4a). They have short, partly stubby (more likely in the small intestine), partly lamellar dendrites (more likely in the colon). In the small intestine, their NF-stained axons start to run preferentially in the oral direction (about 50% vs. 30% anally; Brehmer et al. 2005). Immunohistochemically, they contain choline acetyltransferase (ChAT), leu-enkephalin (ENK) and, partly, SP (Brehmer et al. 2005; Beck et al. 2009). These chemical characteristics correspond to ascending motor and interneurons identified by Porter et al. (1997) and Humenick et al. (2021).

### Spiny type I neurons

With their short processes, these NF-stained neurons also correspond to Dogiel type I neurons. Occasionally, the dendrites have widened, lamellar endings or branching points, but their main appearance is spiny or thorny (Fig. 4b). In contrast to stubby neurons, the dendrites of spiny neurons also frequently emerge from the luminal somal surface, and the whole cell has a hedgehog-like appearance (Lindig et al. 2009). The axons preferentially start running anally; they are immunopositive for neuronal nitric oxide synthase (nNOS) and vasoactive intestinal peptide (VIP; Brehmer et al. 2006), and partly (maybe additionally?) for nNOS and ChAT (Beck et al. 2009). This corresponds to the chemical codes of circular muscle motor neurons (Wattchow et al. 1997; Porter et al. 1997) and descending interneurons (Porter et al. 2002; Humenick et al. 2021), respectively.

### Hairy type I neurons

Uniaxonal, short-dendritic neurons (“*Dogiel type I*”) which project from the myenteric plexus to the mucosa were not included in Dogiel’s classification. These *Stach type IV* neurons were first observed in pigs and guinea pigs (Stach 1982b, 1989; Furness et al. 1985; Brehmer et al. 1999b). Human myenteric, uniaxonal neurons projecting to the mucosa were suggested by Stach et al. (2000) using classical silver staining, while the results of target-specific tracing studies in the human gut were ambiguous (Wattchow et al. 1995; Hens et al. 2001). The latter authors found SOM+/SP± and VIP+ myenteric neurons traced from the jejunal mucosa of four infants. In the human stomach, myenteric neurons innervating mucosal cells were characterized immunohistochemically, and these neurons were found to contain ChAT, VIP, gastrin-releasing peptide and neuropeptide Y (Anetsberger et al. 2018; Furness et al. 2020). Their NF-morphology resembled a “hairy head”, and they had short, extremely thin dendrites (Fig. 4c, Anetsberger et al. 2018). Subsequently, such myenteric neurons co-reactive for NF+/ChAT+/VIP+ were also seen in the small intestine (unpublished observations). A colonic myenteric neuron projecting directly towards the external submucosal plexus is depicted in Fig. 3. It displayed short, thin dendrites, but overall, and in contrast to gastric hairy neurons, it resembled a less hairy head.

### Long-dendritic, uniaxonal neurons

**Fig. 5:**
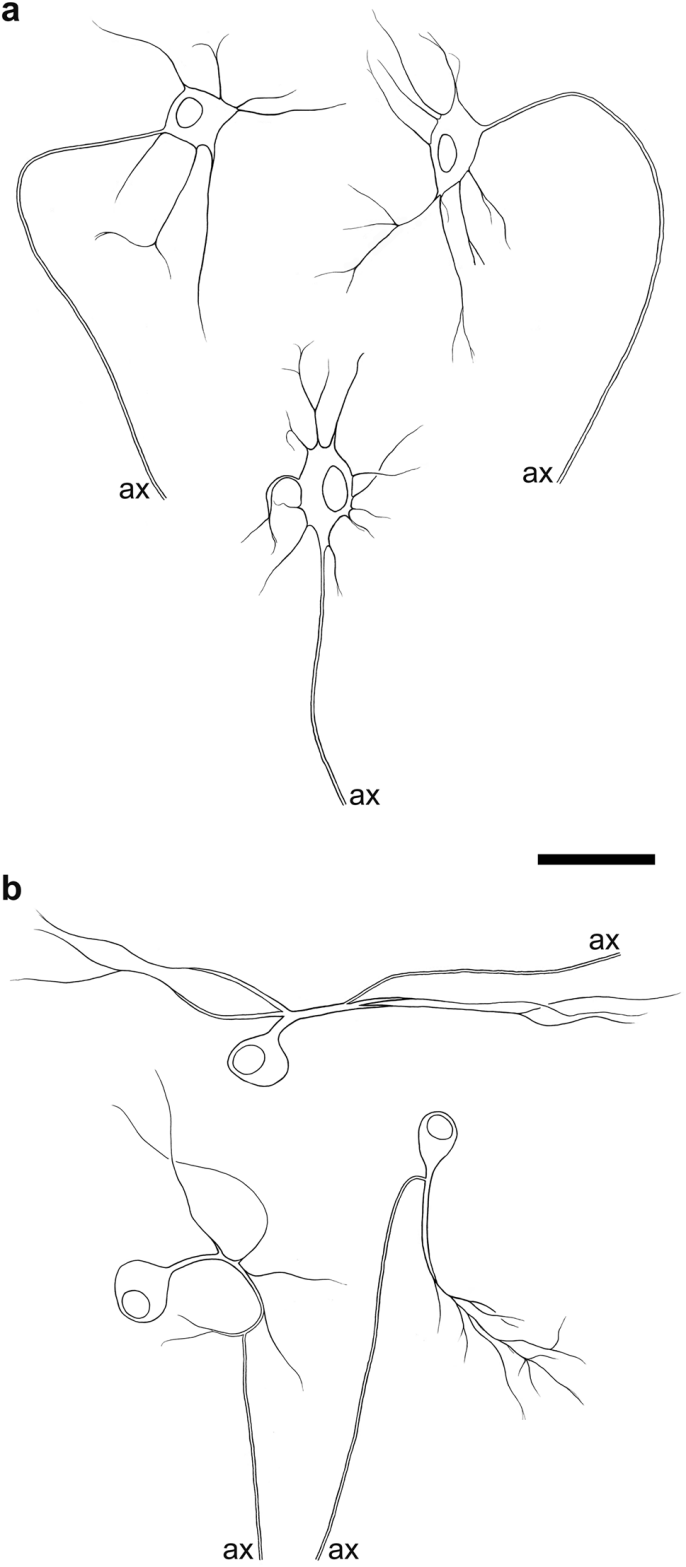
**a** Drawings of three (radial long-dendritic) type III neurons from the small intestine. **b** Three (polar long-dendritic) type V neurons. (ax = cut ends of axons) Bar = 50 µm

Both human myenteric type III and type V neurons have been observed so far, based on silver staining and NF-immunohistochemistry, in the small intestine only (Stach et al. 2000; Brehmer et al. 2004a). Their type-specific immunohistochemical characterization awaits more detailed analysis. Although objectifying morphometric evaluations of enteric dendritic tree patterns are rare (Brehmer and Beleites 1996) and still pending in the human gut, the difference between neurons with short (Fig. 4a–c) and with long dendrites (Fig. 5a, b) is impressive at first glance.

*Type III neurons* (Fig. 5a). Dogiel‘s (1899) depictions of two myenteric type III neurons derived from the guinea pig large intestine. Nearly a century later, type III neurons were rediscovered and precisely described in the pig upper small intestine (Stach 1982a). In humans, their unequivocal representation using NF-immunohistochemistry succeeded especially in the small intestinal myenteric plexus (Brehmer et al. 2004a). These radial long-dendritic neurons are non-nitrergic and mostly cholinergic (Brehmer et al. 2004a; Beck et al. 2009), which is in sharp contrast to pig nitrergic long-dendritic (type III) neurons (Timmermans et al. 1994; Brehmer and Stach 1997). This difference indicates that an appropriate comparative morphology, i.e. the definition of functionally equivalent enteric neurons across species, cannot be established by pure transfer of superficial shape criteria from one species to another. Human type III neurons display immunoreactivity for CALB (Zetzmann et al. 2018), and CALR reactivity was demonstrated in some of them (Brehmer et al. 2004b). Tracing studies in the human colon have assigned neurons with ChAT+/CALB+ and ChAT+/CALR+ immunoreactivity to ascending and descending interneurons, respectively (Humenick et al. 2021). However, on the one hand, long-dendritic neurons in the human colon have not yet been successfully demonstrated by NF-staining (apart from single exceptions; Beck et al. 2009), and on the other hand, a specific chemical code of type III neurons in the human small intestine is not yet defined (Zetzmann et al. 2018).

*Type V neurons* (Fig. 5b). These polar long-dendritic myenteric neurons were first described as a peculiar population in the pig lower small intestine, where they occur in two forms: as single cells and in aggregates (Stach 1985; Brehmer et al. 2002a, b). Their putative functional equivalence with ChAT+/SOM+ co-reactive interneurons of the guinea pig (Portbury et al. 1995) has been discussed (Brehmer et al. 2004b; Brehmer 2006). Human type V neurons, in striking contrast to human “multipolar” type III neurons, appear as “unipolar” neurons mostly displaying a single stem process from which both several long, branched, tapering dendrites and the single axon emerge. With this architecture of processes, they resemble “monopolar invertebrate motor neurons” (Smarandache-Wellmann 2016). Human type V neurons are non-nitrergic and cholinergic; a minority (16%) display additional SOM-reactivity (Brehmer et al. 2004a).

## Regional proportions of myenteric neurons

Overall, myenteric neurons can be immunohistochemically grouped into two large populations, namely neurons reactive for nNOS or for ChAT, as well as into two smaller populations immunoreactive for both or for neither of these markers, respectively. Data based on this categorization were provided for the human stomach by Pimont et al. (2003) and Anetsberger et al. (2018), for the human small intestine by Beck et al. (2009) and for the human colon by Murphy et al. (2007) and Beck et al. (2009).

**Fig. 6:**
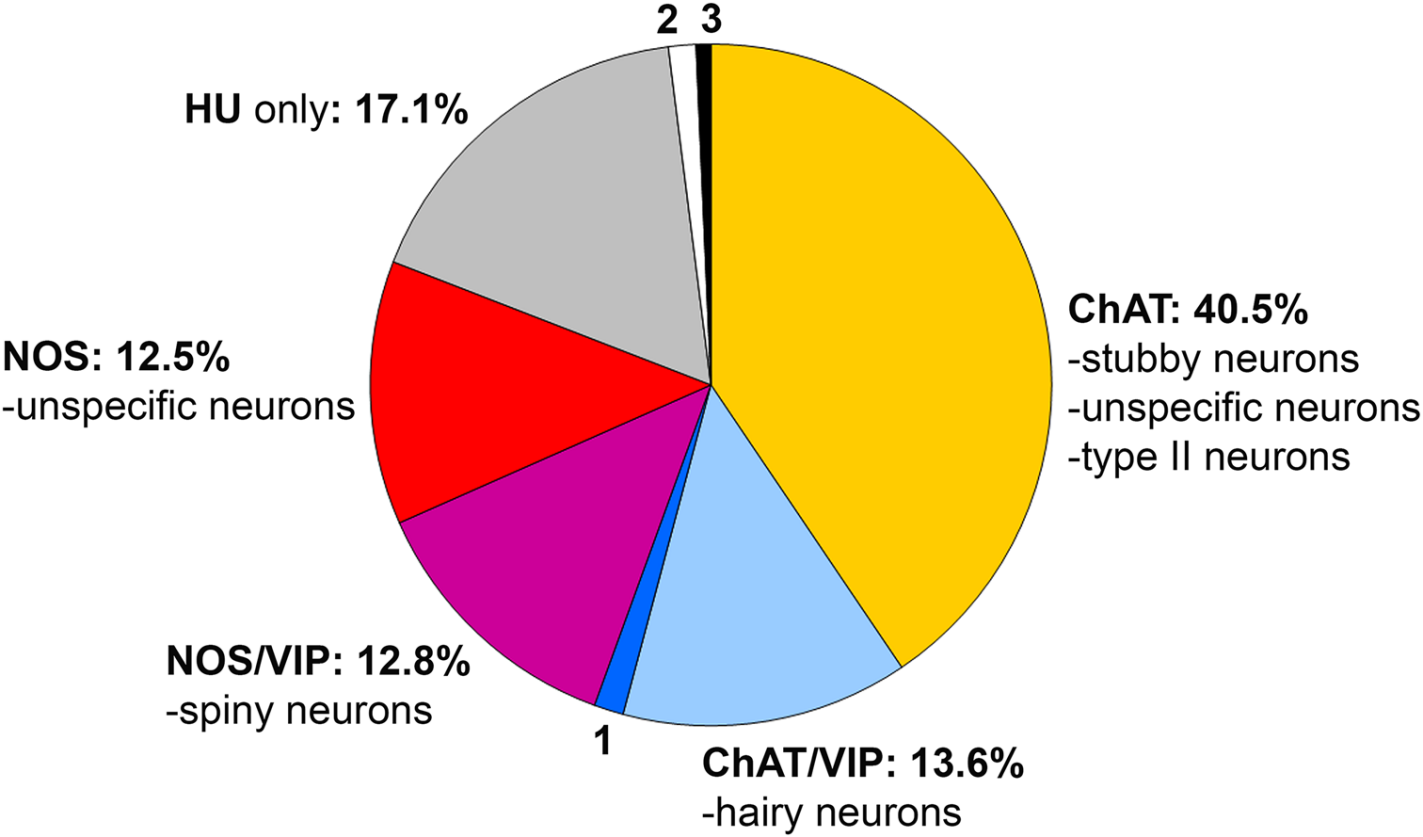
Morphochemically defined myenteric neuron populations in the human stomach (data from Anetsberger et al. 2018). The proportions of cholinergic subtypes were not yet estimated. (1: VIP+ neurons, 1.4%; 2: cChAT+/nNOS+ neurons, 1.3%; 3: cChAT+/nNOS+/VIP+ neurons, 0.7%)

*For the stomach*, data from Anetsberger et al. (2018) are graphically summarized in Fig. 6. Nitrergic neurons included mainly two groups: nNOS+/VIP+ neurons were mostly spiny neurons, whereas nNOS+/VIP− neurons displayed, apart from a few spiny neurons, no specific dendritic architecture (each about 12%). Such morphologically “unspecific”, “simple” or “small” neurons are also known from other species (Stach 1989; Qu et al. 2008). Cholinergic neurons included ChAT+/VIP+ hairy neurons (about 13%) as well as several different ChAT-only neurons (about 40%), namely stubby neurons, “unspecific” neurons without distinct dendritic trees, and a few multiaxonal type II neurons (less than 1%).

**Fig. 7:**
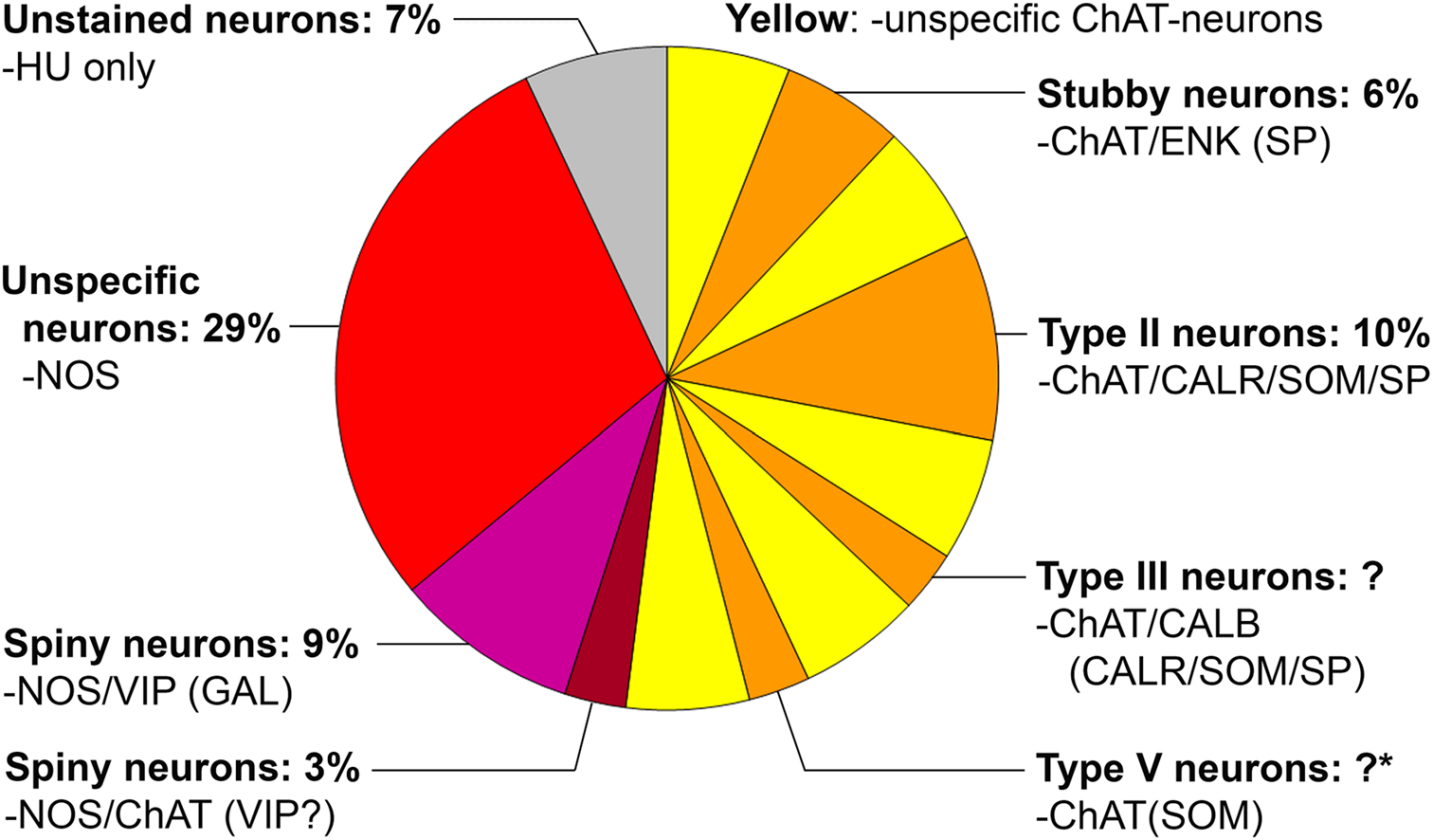
Morphochemically defined myenteric neuron populations in the human small intestine. Undefined cholinergic neurons are illustrated in yellow. Proportions of type III and type V neurons were not yet estimated; the latter are mainly present in the upper small intestine (asterisk). Data from Brehmer et al. (2005, 2006), Weidmann et al. (2007), Beck et al. (2009), Schuy et al. (2011), Zetzmann et al. (2018)

*In the small intestine*, the morphological diversity of myenteric neurons is most pronounced. Besides short-dendritic (stubby and spiny) and non-dendritic type II neurons, long-dendritic neurons are common in this longest gut segment. Type III neurons are present in all small intestinal subregions, while type V neurons are conspicuous mainly in the duodenum and upper jejunum. In Fig. 7, results of several studies were combined to show that the morphological diversity is greatest among cholinergic neurons. These include stubby (type I) as well as type II, III and V neurons. The nitrergic spiny (type I) neurons are depicted as two separate nitrergic populations (+VIP vs. +ChAT), although they may overlap.

*Colonic putative IPANs* were identified by NF-immunohistochemistry. These multiaxonal type II neurons are common in the large intestine (Beck et al. 2009; Zetzmann et al. 2018) but have so far only been focused on in the small intestine in terms of their chemical coding and proportion (Weidmann et al. 2007).

*Colonic interneurons* were identified by DiI-tracing, with four ascending and descending populations each distinguished (Humenick et al. 2021). Among the ascending populations, three contain ChAT and ENK and correspond immunohistochemically to stubby neurons (Brehmer et al. 2005). Among the descending populations, ChAT+/nNOS+ interneurons chemically correspond to spiny neurons (Beck et al. 2009), whereas nNOS-only neurons may be unspecific neurons without conspicuous dendritic trees. Colonic ChAT+/CALB+ ascending or ChAT+/CALR+ and ChAT+/5HT+ descending interneurons have not yet been identified as to their NF-morphology.

*Colonic circular muscle motor neurons* included ascending, ChAT+ neurons and descending nNOS+/VIP+ neurons (Porter et al. 1997), the latter corresponding to nNOS+/VIP+ spiny neurons (Brehmer et al. 2006).

*Colonic longitudinal muscle motor neurons* included ChAT+ ascending as well as nNOS+ and nNOS+/VIP+ ascending and descending neurons (Humenick et al. 2019). nNOS+/VIP+ and nNOS+/ChAT+ spiny neurons, from stomach to colon, may be overlapping populations (we have observed neurons co-reactive for nNOS, VIP and ChAT but not yet estimated quantitatively). They may be both inter- and motor neurons.

Colonic and small intestinal myenteric neurons projecting outside the muscle coat have to be further investigated. By DiI-tracing, such neurons were identified by Wattchow et al. (1995) in both the small and large intestine as well as by Hens et al. (2001) in the jejunum of infants. These may include type II neurons with axons running towards the submucosa (Brehmer et al. 2004b) as well as hairy neurons (subtype I of Dogiel, type IV of Stach) in the stomach (Anetsberger 2018) and in the colon (Fig. 3).

## Structure of human submucosal neurons

**Fig. 8:**
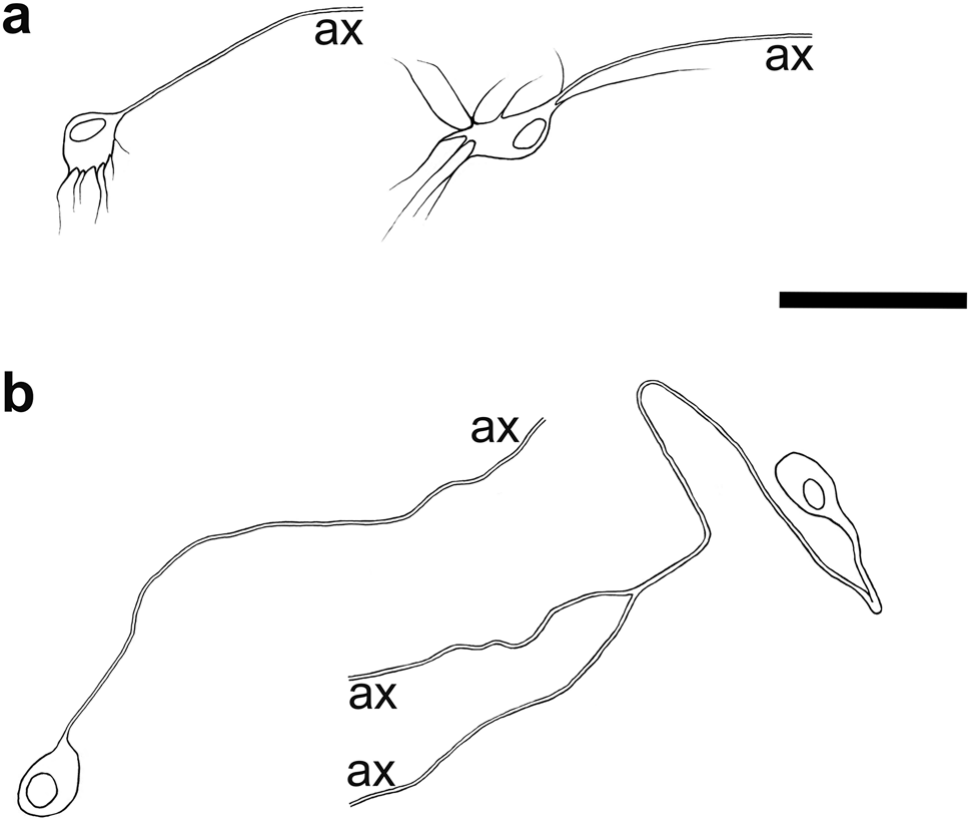
**a** Drawings of two dendritic submucosal neurons. **b** Two non-dendritic submucosal neurons with one axon. The axon of the right neuron could be observed until its branching point. (ax = cut ends of axons) Bar = 50 µm

Historically, the first categorizations of submucosal neurons were published by authors other than Dogiel (Kustermann et al. 2011). These were Koelliker (1896) in the dog, Ramón y Cajal (1911) in the guinea pig, Rossi (1929) in the pig, Sokolova (1931) in cattle and Stöhr (1949) in humans. A general structural dichotomy into multipolar and unipolar (and occasionally also bipolar) neurons was detected and could be, to some degree, also correlated with the chemical codes of neuron populations in human small and large intestinal submucosal neurons (Fig. 8, Table 2).

**Table 2:** Summary of human submucosal neuron types based on their morphological properties observable after immunostaining for peripherin (PERI)

Submucosal neuron type	Basic morphological description	Axon projection Dendritic architecture	Chemical coding	Main function?
Unipolar (rarely bi- or tripolar)	Pseudouniaxonal Non-dendritic	A: to mucosa?	ChAT/SOM/SP±	Mucosal afferent neuron?
Multipolar	Uniaxonal Dendritic	A: to mucosa? D: slender, short to medium length	ChAT/CALR±/VIP	Mucosal effector neuron?
(Nitrergic neurons)	Not determined	?	nNOS	Circular muscle motor neuron?

For details and references see text

Although the human submucosal plexus, similar to that of other medium-sized mammals, consists of two topographically distinct networks (reviewed in Brehmer et al. 2010), there seem to be only quantitative differences in neuronal composition between them (Jabari et al. 2014). This is in striking contrast, for example, to the pig submucosa, where qualitative differences between the external and the internal submucosal plexus (ESP, ISP) are conspicuous (Stach 1977; Timmermans et al. 2001; Kapp et al. 2006; Petto et al. 2015). The demonstration of human submucosal neuron morphology was more successful using PERI- instead of NF-antibodies (Ganns et al. 2006; Kustermann et al. 2011).

### Submucosal unipolar (pseudouniaxonal) neurons

These neurons (Kustermann et al. 2011) have a small round or oval cell body with a single (seldom two or three) process, most likely an axon. Occasionally, the process could be traced until its branching point; thus these neurons may be pseudouniaxonal. Immunohistochemically, they contain ChAT, SOM and, partly, SP (Beyer et al. 2013). Based on the structure and the localizations of ChAT+/SOM+/SP+ endings in the mucosa, we suggest a sensory function. To some extent, the identification of mechanosensitive ChAT+/SP+ neurons (Filzmayer et al. 2020) supports this assumption.

### Submucosal multipolar (dendritic, uniaxonal) neurons

In contrast to the unipolar neurons described above, these obviously have numerous processes, most of which being dendrites (Kustermann et al. 2011). Based on their PERI-staining, we observed these neurons to be mostly uniaxonal (seldom bi- or tripolar), a finding supported to some extent by the descriptions by Porter et al. (1999). Furthermore, they are co-immunoreactive for ChAT, CALR and VIP. It has to be considered that the colocalization rate of CALR and VIP is almost 100% in colonic submucosal neurons, whereas in the small intestine, up to 16% of VIP-reactive neurons do not co-stain for CALR (Beuscher et al. 2014). Boutons co-stained for VIP and CALR were found exclusively in the colonic mucosa (Beuscher et al. 2014); therefore, a mucosal effector function has been suggested (Jabari et al. 2014). Contrary to these results, Porter et al. (1999) found VIP+ submucosal neurons projecting to the circular muscle.

### Submucosal nitrergic and other neurons

nNOS+ neurons represent a small submucosal population (1–4%; Beuscher et al. 2014), and an even smaller one is represented by nNOS+/VIP+ colocalization (max. 1%, in the external submucosal plexus; Beuscher 2014; Porter et al. 1999). It is supposed that these neurons are dendritic and uniaxonal, though this has not yet been investigated in detail.

Moreover, Beuscher et al. (2014) found that more than 30% of small intestinal and more than 10% of colonic submucosal neurons were only stained by HU. Thus, there may be three or more populations in the human submucosa as found in the mouse (Wong et al. 2008; Mongardi Fantaguzzi et al. 2009) and guinea pig (Furness et al. 2003), respectively, which remain to be further characterized.

## Regional proportions of submucosal neurons

*In the stomach*, no continuous submucosal plexus could be found and, as compared with the intestines, very few submucosal neurons were present (Anetsberger et al. 2018). Morphologically, both neuron types (non-dendritic and dendritic, see above) were identified, although no clear morphochemical correlation could be proved.

*In the intestines*, so far, we have found no qualitative differences in neuronal composition, either between the small intestine and colon or between the ESP and the ISP (Kustermann et al. 2011; Beyer et al. 2013; summarized and graphically illustrated by Beuscher et al. 2014). Basically, four populations, three larger and one smaller, were identified. All of them may include subpopulations. The most striking difference between the small intestinal and the colonic submucosa is the proportion of VIP+ neurons, though it has to be emphasized that the variability is enormous.

*VIP*+*/ChAT*±*/CALR*± *dendritic uniaxonal neurons* amounted to 32–39% (ISP-ESP) in the small intestine and 72–74% in the colon.

*SOM*+*/ChAT*+*/SP*± *pseudouniaxonal neurons* accounted for 36–24% (ISP-ESP) in the small intestine and 14–10% in the colon.

*nNOS*+ *neurons* were between 1 (ISP of both segments) and 4% (colonic ESP). Here, up to 1% nNOS+/VIP+ neurons were additionally identified.

*Immunohistochemically uncharacterized (HU*+ *only) neurons* amounted to 11–34%.

## Epilogue: from present to future

Here, we tried to emphasize the importance of morphological analysis of the processes of human enteric neurons. Dendritic architecture is one cornerstone for evaluating synaptic connectivity, and axonal projection is more than a symbolic link to the function of a neuron. Both the omnipresence of, e.g., stubby and spiny type I neurons from stomach to colon and the limited occurrence of, e.g., type V neurons only in the upper small intestine indicate general principles as well as local characteristics of the neuronal composition of the human ENS. Such regional differences should also exist in the human colon, as indicated by differences in motor patterns between the upper and lower colon in the mouse (Costa et al. 2021). The further classification of human enteric neurons must also consider regional peculiarities. This is a basic requirement for understanding physiological functions, pathological processes and, ultimately, options for therapeutic interventions in different regions of the gastrointestinal tract. To this end, all methodical approaches available may contribute, both “classical” and “modern” ones.
